# ChemChains: a platform for simulation and analysis of biochemical networks aimed to laboratory scientists

**DOI:** 10.1186/1752-0509-3-58

**Published:** 2009-06-06

**Authors:** Tomáš Helikar, Jim A Rogers

**Affiliations:** 1 Department of Pathology and Microbiology, University of Nebraska Medical Center, 983135 Nebraska Medical Center, Omaha, NE 68198, USA; 2 Department of Mathematics, University of Nebraska, 6001 Dodge Street, Omaha, NE 68182, USA

## Abstract

**Background:**

New mathematical models of complex biological structures and computer simulation software allow modelers to simulate and analyze biochemical systems *in silico *and form mathematical predictions. Due to this potential predictive ability, the use of these models and software has the possibility to compliment laboratory investigations and help refine, or even develop, new hypotheses. However, the existing mathematical modeling techniques and simulation tools are often difficult to use by laboratory biologists without training in high-level mathematics, limiting their use to trained modelers.

**Results:**

We have developed a Boolean network-based simulation and analysis software tool, ChemChains, which combines the advantages of the parameter-free nature of logical models while providing the ability for users to interact with their models in a continuous manner, similar to the way laboratory biologists interact with laboratory data. ChemChains allows users to simulate models in an automatic fashion under tens of thousands of different external environments, as well as perform various mutational studies.

**Conclusion:**

ChemChains combines the advantages of logical and continuous modeling and provides a way for laboratory biologists to perform *in silico *experiments on mathematical models easily, a necessary component of laboratory research in the systems biology era.

## Background

As our understanding of cellular processes such as signal transduction, genetic regulation, etc., grows, it is becoming clear that their emerging complexity means that they can no longer be studied exclusively by classical reductionist techniques [1]. Thus, a systems approach is required if these cellular functions are to be fully understood [1,2]. A systems approach to the study of biochemical networks requires the creation of models (and software to simulate them) that take into account the numerous interactions of chemical components of the whole system [2,3]. The subsequent use of these models and software tools have the potential to serve laboratory biologists as a complimentary method to pre-screen their laboratory experiments, as well as help them to refine or even develop new hypotheses.

The most common ways to represent these interactions are ones using continuous methods, generally including ordinary differential equations (ODE) or partial differential equations (PDE) [2,4-6]. To put them in motion, a number of software tools to simulate and subsequently analyze the dynamics of these models have been developed (e.g., E-Cell [7], CellDesigner [8], Dizzy [9], Cellerator [10,11], Virtual Cell [12], *etc*.). However, one of the problems with using differential equations is that each equation requires the knowledge of many parameters that make up the kinetic basis of the network interactions, which in many cases (especially for large-scale models) may be difficult to obtain [3]. In addition, as the size and connectivity of the models increases, the complexity of the underlying differential equations also increases, limiting their use to only investigators trained in higher-level mathematics.

An alternative to differential equation modeling is the use of discrete models [3,13]. This method is based on qualitative and parameter-free information (e.g., protein *x *activates protein *y*) which is available in the biomedical literature and/or directly from laboratories, simplifying the process of building and modifying the models. Although discrete (Boolean) models have been adopted to study the dynamics of gene regulatory networks and in the studies of signal transduction networks [14-16], the overall use of Boolean models to visualize biochemical processes is sparse relatively to the differential equation-based approach. As a result, only a limited number of software tools based on this approach exist (e.g., GinSim [17], SQUAD [18], and CellNetAnalyzer [13]).

One reason for the lack of development of Boolean modeling tools for life sciences is that biologists aren't well versed in discrete modeling. In most cases, nodes in such models are in either an ON or OFF state, often represented by '1' and '0', respectively. For laboratory scientists who are accustomed to dealing with continuous data (e.g., dosage levels, protein activity levels, etc.), such representation may be unintuitive and difficult to use. Thus one way to advance the use of discrete models for biological systems would be to create the ability to interact with them using continuous terms.

In this report, we describe in detail ChemChains, a suite of software tools used in our recent study [14]. ChemChains was developed as a core platform to simulate, analyze and visualize the dynamics of large-scale Boolean biochemical networks under tens of thousands of different environments, while enabling users to interact with the model in a continuous manner. Thus biological investigators can interact with their models in a familiar way, while preserving the benefits of parameter-free models. Although ChemChains simulations performed in [14] were done in a synchronous fashion (i.e., all nodes in the model updated at the same time during every simulation step), ChemChains also offers asynchronous updating where certain nodes can update at different time points during the simulation process.

## Implementation

### Boolean networks and their dynamics

Although relatively simple, Boolean networks are able to capture the dynamics of systems ranging from trivial to exceedingly complex, including those of living systems [19].

#### Boolean networks

**Figure 1:**
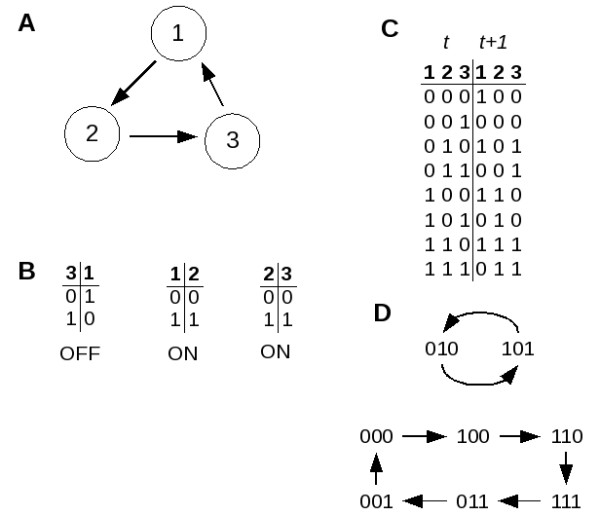
**A simple network and its logical connections**.

These networks are collections of labeled Boolean nodes connected with directed edges. In Boolean networks, the state of each node at time *t *can be either ON or OFF, often represented by '1' and '0', respectively. Consider the simple network shown in Figure 1A[20]. This network has three nodes and each node is connected to each of the others. The activation function (or mechanism) and the logical connections for each node can be described by a truth table (Figure 1B). Truth tables give the ON/OFF state (shown as a 1 for on and a 0 for off) of each node as a function of the ON/OFF state of the other two nodes connecting to it. Herein, nodes that determine the activation function of another node are referred to as "input nodes" or "inputs". Thus the third table shows that node 3 will be on if either input 1 or 2 (or both) are on.

The network exists at time *t *in some initial state, with each separate node either on or off. At the next time (*t *+ 1), the states of all three nodes will change according to the tables shown. The evaluation of the entire system from time *t *to time *t *+ 1 can be represented in a single table (shown in Figure 1C) where the column *t *contains all the possible initial states of the system and column *t *+ 1 shows the result of application of the logic set to each initial condition. Continued iteration by the same method results in a trajectory of the system as the states change over time.

#### Boolean attractors

**Figure 2:**
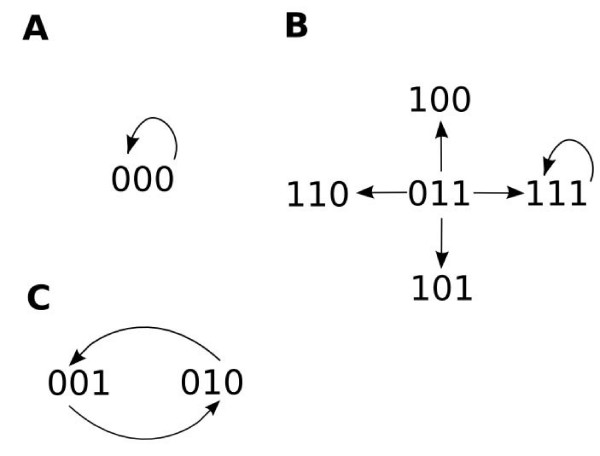
**All possible trajectories and attractors for the network in Figure 1**.

The network introduced in Figure 1 is simple enough to view all of the possible trajectories, which are shown in Figure 2[20]. In panel A, for example, the system is shown to be at an initial state of node 1 = 0, node 2 = 0 and node 3 = 0, or 000. According to the logic tables in Figure 1B (or, equivalently, the map shown in Figure 1C), at the next time point the system will remain at 000. This trajectory is indicated by the arrow in Panel A. Similarly, Panels B and C show the trajectories for the other possible starting combinations.

Because there are a finite number of nodes in the system (N), there are a finite number (2^*N*^) of possible states of the system. Thus, as the system travels in time, it must (regardless of trajectory) re-enter a state previously encountered. As shown in Figure 2A and 2B, when the system is at state 000 or 111, it remains there (encountering itself over and over), thus those two states are referred to as steady-states. Panel C shows that if the system is at state 001 or 010, it cycles between those two states, a trajectory that is referred to as a period 2 cycle. Finally, panel B shows that there are four other states of the system (110, 100, 011, and 101) that follow trajectories to the steady-state 111. In summary, Figure 2 shows that the network described in Figure 1 has three conditions (namely the two steady-states 000 and 111, and a period 2 cycle containing 001 and 010) called attractors into which the trajectories of all initial states eventually settle.

#### Using attractors to characterize node activation

**Figure 3:**
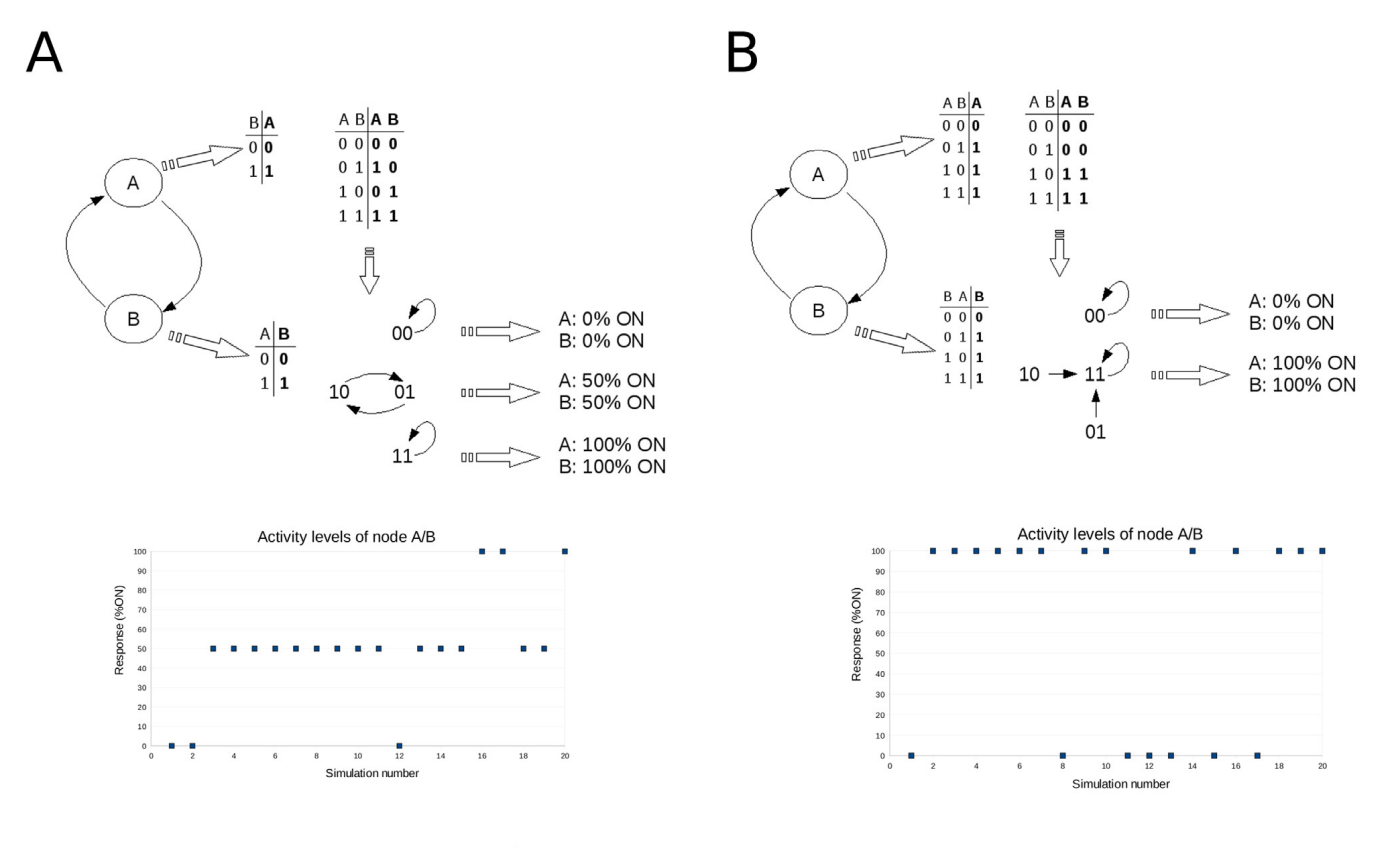
**A simple positive feedback loop**. Two possible configurations of a positive feedback loop in which node A activates node B and node B activates node A. A) First configuration, in which the truth tables and state transition diagram depict the most trivial activation mechanisms of nodes A and B; each node is simply activated when the other node is 1 and deactivated when the other node is 0. In addition a sample simulation experiment (consisting of 20 simulations with randomly selected initial states) using ChemChains was done to illustrate the connection between the system's three attractors and the percent ON measure of each node (note that the percent ON levels where the same for both nodes, hence one diagram per configuration is shown). B) In the second sample configuration of a two-component positive feedback loop the truth tables depict activation mechanisms of both nodes in which each node is, similarly to the first configuration, activated by the other node, but is not necessarily turned off when the other node is off. As illustrated by the state transition diagram and a ChemChains simulation experiment (also consisting of 20 simulations with randomly selected initial states) in Panel B, this set-up results in two attractors, and thus two possible percentage ON levels (0% and 100% ON) for each node, depending on the initial condition.

As can be seen in Figure 2, once a Boolean network has settled onto an attractor, it will remain there. Thus, it is possible to characterize the activation state of each node in the network by determining the "percent ON" of each node over the entire attractor. For example, there are three attractors (Figure 2) of the network introduced in Figure 1. Looking at the fixed point attractors in Panels A and B in Figure 2, it is obvious that all three nodes are 0% and 100% ON, respectively. However, in Panel C there are different behaviors. On this attractor, node 1 is 0% ON, but nodes 2 and 3 are both 50% ON as they alternate between 0 and 1 at every step in the attractor. Consider another example – a simple hypothetical two-component positive feedback loop in which node A activates node B and vice versa with two different configurations of truth tables for each node (Figure 3A &3B, respectively). In Figure 3A, both nodes are activated when the other node is 1, but deactivated when the regulating node is 0. As shown in Figure 3A, this configuration and activation mechanism results in three attractors (or tristability), that will make both nodes either 0%, 50%, or 100% ON, depending on the initial state of each node. The tristability (in terms of percent ON) is also demonstrated by results from a sample ChemChains simulation experiment shown in the graph in Figure 3A (bottom). The second truth table configuration (Figure 3B) depicts an activation mechanism in which both nodes lack the negative regulatory step (where the inactive state of the regulating node causes the other node go to 0). As shown in Figure 3B, setting up the truth tables this way results in the system moving to one of two attractors, depending on the initial condition (bistability). In terms of percent ON, this configuration results in both nodes being either 0% or 100%ON as shown by results from a ChemChains simulation experiment (Figure 3B). It is also worth noting that bistability (demonstrated in the above example) is an intriguing higher order property of positive feedback regulation found in many real biological networks. The Boolean positive feedback loop in Figure 3B hence makes a nice example of how the bistability phenomenon can be simulated using ChemChains.

### ChemChains overview

ChemChains is a platform-independent, command-line-driven simulation and analysis tool for Boolean networks implemented in C++, using object-oriented methodology. By default, ChemChains uses synchronous updating for all nodes in the model, however, asynchronous updating for user-selected nodes is also available (see Delay and Sustain Nodes sections below). The software is built in an extension-based fashion, which will allow for the expansion of the collection of analysis tools used in ChemChains. The current version of ChemChains consists of the main simulator engine and two extensions (described below) used in our recent publication on information processing in signal transduction networks [14]. To run ChemChains, users must provide several input files (described below). Once simulations and/or analyses are initiated, ChemChains will create several types of output file formats. Both the input files, as well as the desired output format and the output files must be specified by users in the form of command-line parameters provided to ChemChains at the time of program initiation. Available parameters are summarized in Table 1.

**Table 1:** Summary of available ChemChains parameters.

**Parameter**	**Description**
***General parameters used with all running modes***	
-ispecs *filename*	Simulation specification file name
-ilogic *filename*	Network descriptor file name
-v	Verbose mode
***Parameters used with FileConversion mode***	
-TT2ND	Creates a network descriptor file from a set of truth tables
-ND2TT	Converts the network descriptor into a set of truth tables
-itables *path*	File path for list of truthtables, and the nodelist
-inodelist *filename*	File for List of Nodes, Used with TT2ND
-o *filename*	Specifies the location of FileConversion output
***Parameters used with Visual mode***	
-vis *filename*	Instantiates ChemChains in the visual mode and saves the output file provided by *filename*
-A	Output all nodes
***Parameters used with Calculation mode***	
-calc *experimentname*	instantiates ChemChains in the calculation mode
-n *xxx*	Number of consecutive simulations
-noBits	Suppress printing of bit files
-rand_init	randomly selects initial states for all nodes and creates new logic file
***Parameters used with Pattern analysis extension***	
-patterns	runs pattern analysis after each simulation
-isettings *xxx*	Patterns file with node activity range settings
-inodes *xxx*	File with nodes to be analyzed

### Software input

To simulate Boolean networks with ChemChains, two input files are required: a network descriptor file and a simulation specification file.

#### Network descriptor

Network descriptor file is a ChemChains-specific text file containing the collection of activation mechanisms of Boolean nodes in a given Boolean network model. In this file, the activation mechanism of each node is described on a new line in the following format:

Bool:name:initial state(True or False):input1, input2..inputN:list of states of input nodes for non-initial state of a node of interest (separated by comma)

The "Bool" prefix tells the software that the node is of type Boolean (users can also declare other types of nodes such as Input, Sustain, Delay, and Output, all of which are discussed below).

For example, consider a node X with two input nodes A and B that both need to be ON in time t for X to be ON in t+1. The truth table depicting this scenario (Table 2) is represented as (initial state of X = ON):

**Table 2:** Truth table for node X

A	B	**X**
1	1	**1**
1	0	**0**
0	1	**0**
0	0	**0**

*Bool:X:True:A, B:TF, FT, FF (i.e., node X with inputs A and B is ON, unless A and B, in time t, are: A-ON & B-OFF, A-OFF & B-ON, or A and B are both OFF)*,

or for the case where the initial state of X is OFF:

Bool:X:False:A, B:TT (i.e., node X with inputs A and B is OFF, unless A and B, in time t, are both ON)

The initial state (i.e., *t *= 0) of each node has to be defined prior to any simulations. The initial states can be assigned either by the user (see the "FileConversion" section below), or for more general analysis, randomly by ChemChains using the 'rand_init' parameter. Using the 'rand_init' parameter results in the creation of a new network descriptor file during each simulation, reflecting the new initial states of all nodes. Each network descriptor file is subsequently saved in the 'logic' folder (output formats and directories discussed below) of the conducted experiment for later review.

#### Simulation specification file

ChemChains is a feature-rich logic network simulation software which offers users many advanced simulation options. These options are specified by users in the simulation specification file, the second of the required input files, which is loaded into the program before each simulation experiment. The following describes the various options contained within this file:

##### a) Runtime

The runtime variable specifies the overall length of each simulation, as well as the number of iterations (transient time) before any analysis is conducted. The syntax for the runtime environment is specified as

RunTime:TransientTime:TotalTime

For example, to conduct a simulation consisting of 200 iterations and 50 transient time units, the runtime is declared as

RunTime:50:200

##### b) External Input nodes

ChemChains users have the ability to specify external input nodes (or "external inputs") representing various external biological factors (such as receptor ligands, stress, *etc*.). Note, that external input nodes are not the same as the "input nodes" (or "inputs") discussed in the *Boolean networks *section. In fact, external input nodes are external to the network and part of the outside environment. Although they are of a Boolean nature (*i.e*., can be either 0 or 1), external input nodes do not have any input nodes of their own (and hence no truth table). In fact, their activity is set by the user (or randomly selected by ChemChains as shown below). To provide users the ability to interact with the logical model in a continuous manner, the level of activity of each external input node can be set to a percentage ON (specified by an integer 0–100). Despite the fact that external input nodes are Boolean nodes, for any given simulation, this is accomplished by placing the external input nodes on a cycle that yields a desired % ON (see the *Using attractors to characterize node activation *section). For example, if a particular external input is set at 33%, it is put on a cycle of 100100100100... which is held constant for the duration of the experiment. The sequence and order of the ON- and OFF- states is generated by ChemChains during each simulation. The current implementation of ChemChains offers two ways to distribute the ON/OFF state along this sequence: i) evenly (i.e., from the example above, the input will be ON every other iteration) and ii) with noise (the Noise variable is explained in the following section) which allows for a more realistic simulation of a biological process where network inputs are almost always noisy. Users can select time frames of simulations with different levels of activity. For example, in a simulation consisting of 200 iterations, a user may want to see how the network behaves when an input is set to 10% ON during the first 50 iterations, 50% during iterations 51 – 100, and 100% for the remaining number of iterations. Note that the changes in the external input activities occur in a stepwise manner (e.g., as soon as the simulation reaches iteration 51, the activity of an external input will change from 10% to 50%). The format underlying the declaration of external inputs is as follows:

Input:name:initial/default value:random(R)/fixed(F):noise(N)/fixed/(F):time to be introduced:dosage (in percentage):duration of the dosage

Consider the above mentioned example of a 200-iteration network simulation. To set a hypothetical external input "In1" to be ON 10% (with no noise) for the first 50 iterations, 50% ON during iterations 51 through 100, and 100% during iterations 101 – 200 (and with an initial state set to "False"), the following line is added to the simulation specification file:

Input:In1:False:F:F:1:10:49,51:50:49,101:100:99

To run ChemChains in an automated fashion and simulate a network under a wide variety of external environments (by using the -n parameter to run *n *number of consecutive simulations), users can also specify an activity range for a given external input. During each simulation, ChemChains will select a different percentage ON for the external input. For example, to simulate a network with In1 varying between 0 and 100%, In1 must be declared as follows:

Input:In1:False:R:F:1:0-100:199

##### c) Noise

The noise variable allows the activity level of external input nodes to randomly bounce around the defined value within a specified range. For example, if the activity level for an external input is set to 50% with a noise range of 5, the actual activity level, at time *t*, will be 50%+/-5. To measure the current level of activity for each external input, and ensure that it is within the desired range during a simulation, ChemChains uses a "sliding window" (of user pre-defined length) approach. Upon each iteration, the algorithm randomly selects a new activity level value within the given range. If the percentage within the window is below the new value, the external input will turn ON. On the other hand, if the percentage is above the activity level, the external input will be OFF in time *t*+1.

Although the sequence of 0's and 1's for a given external input node will will yield 50% 1's, it will no longer be periodic, instead it will appear chaotic, a more realistic resemblance of biological stimuli. For example, a simulation with the noise parameter set to 5 and a sliding window of length 50 is specified in the specification file as

Noise:50:5

##### d) SnapshotAnalysis

The SnapshotAnalysis parameter allows ChemChains to capture the state of the network dynamics at a specified iteration point during a simulation. The SnapshotAnalysis parameter is specified as follows:

SnapshotAnalysis:period:point1, point2,..pointN

where the *period *defines the length (in number of iterations) of the simulation segment to be analyzed, whereas *point1, point2,...pointN *represent at which iteration the analysis will begin.

##### e) Mutation

The Mutation parameter enables users to turn ON/OFF any node in the network to perform mutagenesis studies (e.g.., gain-of-function, and/or loss-of-function). When a node is "mutated", its corresponding truth table is ignored by the simulation engine and the activity of the node becomes immutable for the entire course of the simulation. For example, to simulate a loss-of-function of node X, the user would declare the Mutation parameter as

Mutation:OFF:X

To make node X constitutively active, 'OFF' is replaced with 'ON'.

##### f) Delay Nodes

While ChemChains simulates networks synchronously by default, it is possible to introduce an asynchronous factor into the simulations by declaring a Delay and/or a Sustain Node (see below for the definition of Sustain Nodes). A Delay node receives a signal from a single network node but will not respond to it for a specified number of iterations. Delay nodes can be created in the simulation specification file in addition to the regular (of type "Bool") network nodes declared in the network descriptor file. The syntax to declare a Delay node is as follows:

Delay:name:initial value:input:iterations to wait

##### g) Sustain Nodes

Similarly to Delay nodes, Sustain nodes receive a signal from a single parent node, but contrary to Delay nodes, Sustain nodes remain in their current state for a user-specified number of iterations, regardless of the state of their inputs. To declare a node as a sustain node in the specification file, the users use the following syntax:

Sustain:name:initial value:input:duration (in iteration units)

##### h) Output Nodes

In addition to "Bool", "(External) Input", "Delay", and "Sustain" nodes, users can create nodes of type "Output". Output nodes mirror the activity of their input node and are used for nodes that will be outputted during the visual mode (discussed below). Output nodes are created in the simulation specification file as follows:

Output:output name:initial value:input node name

### Output formats

Once ChemChains is initiated, a main output directory 'CCoutput' is created outside of the ChemChains program's directory, which subsequently stores all output files generated by ChemChains. Currently, ChemChains can be run in three modes, each producing different types of output formats. The first mode is a file conversion mode used to convert truth tables to a network descriptor file, whereas the second modes is a single-simulation visual mode which allows users to visualize the activity of any given output node during the course of a simulation. The third mode is a calculation mode which enables users to perform automated experiments involving tens of thousands of independent simulations with various levels of network stimuli. All available modes are detailed below.

#### File Conversion mode

As described above, to run ChemChains, a logical network descriptor is required. While it can be created manually, creating truth tables in a tabular form poses a more intuitive way of defining the activation mechanism for each node. Once users create all truth tables describing their network, the truth tables need to be converted to the required network descriptor file. This conversion can be accomplished by using the 'FileConverter' extension available in ChemChains. To use this extension, users can save the truth tables in tab-delimited text files (one truth table per file) and create a list of nodes contained in the network. To initiate the conversion process, ChemChains needs to be run with the '-TT2ND' parameter. In addition, a node list file (Table 3), an output file, as well as a directory path containing the set of truth tables must be specified via the '-iNodes', '-o', and '-iTables' parameters, respectively. Once the conversion is complete, the network descriptor is saved in the previously specified output text file.

**Table 3:** Sample node list file.

**Node Name**	**Node ID**	**Initial State**
Node 1	1	1
Node 2	2	
Node 3	3	1
Input 1	IN4	
Input 2	IN5	

The node list file is a tab-delimited text file is supplied to ChemChains in order to convert a set of truth tables to a network descriptor file. The first column contains the name of all network nodes (including external external inputs). The second column provides the node ID. Note that for external input nodes, the ID has to contain the 'IN' prefix which helps the software to recognize external input nodes from the remaining network nodes. The initial state column is where the user can specify the initial state for each node (while external input nodes are also included in this file, their initial states do not need to be specified, as the sole purpose of this file is to mediate the creation of network descriptor file which solely contains nodes of type "Bool").

Furthermore, FileConverter can be used to re-create the truth table for each node from a given network descriptor file. This is done by instantiating ChemChains using the '-ND2TT' parameter, whereas the input file (network descriptor file) is specified via the '-ilogic' parameter. The corresponding truth tables are created in an output folder defined by the user (by using the '-o' parameter).

#### Visual Mode

**Figure 4:**
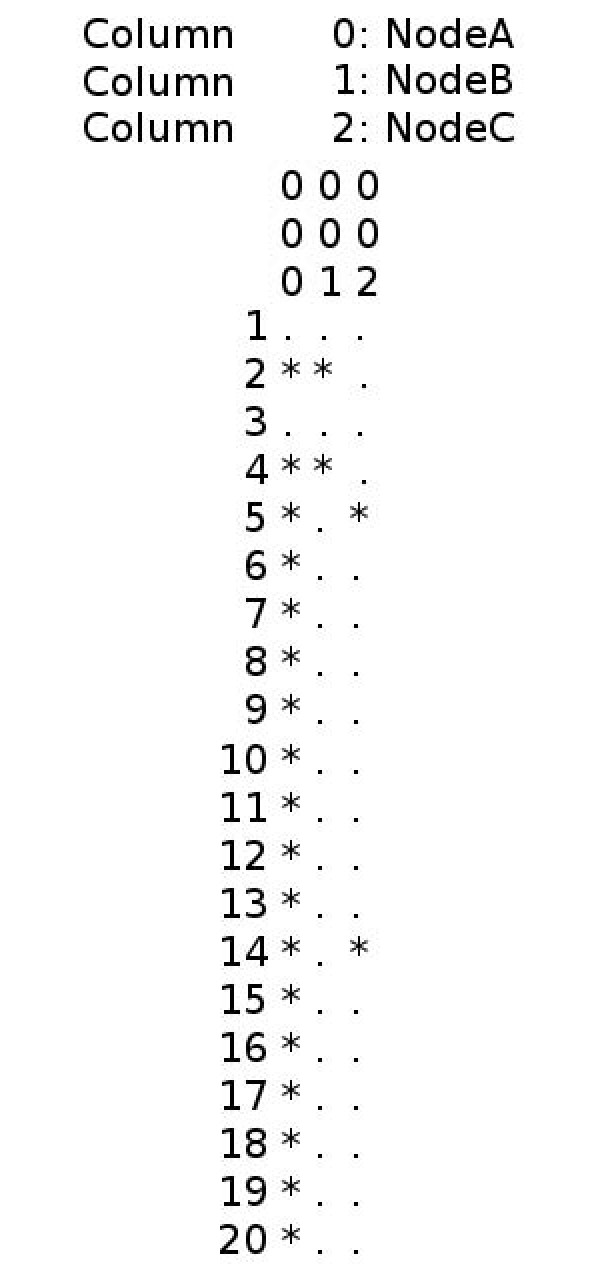
**A sample output file created in the visual mode**. A sample network was iterated 20 times and the activity of three output nodes (NodeA, NodeB, and NodeC) were captured.

The visual mode allows users to visualize the dynamics (*i.e*., the sequence of the ON/OFF states) of the output nodes declared in the specification file (discussed above) during the course of a single simulation. The output created by ChemChains is a text file containing a table of activity for each output node in terms of the OFF/ON states (Figure 4), where '*'(star) represents the ON state, whereas '.'(dot) represents the OFF state of a particular output node. To run ChemChains in the visual mode, the user needs to provide both input files (network descriptor and simulation specification files), as well as an output file using the '-vis' command-line parameter (Table 1). The output file with stars and dots for each output node will be created and saved in the ChemChains output directory.

#### Calculation mode

Once ChemChains is started in the calculation mode, a 'stats' directory is created in the main ChemChains output folder to house all of users' subsequent simulation experiments. Output files of each experiment are stored in the stats directory in a folder labeled by the user (see '-calc' parameter in Table 1). The list of all output files created during each experiment is summarized in Table 4.

**Table 4:** Summary of output files/directories created for each ChemChains experiment.

**File/Directory Name**	**Description**
allNodes_avg.mtb	Tab-delimited text file with activation levels of all nodes across all simulations
input_dosages.csv	Activity levels of all external inputs across all simulations
patternNodes_avg.csv	Activity levels of nodes specified for pattern analysis. (Created only during pattern analysis)
input_labels.csv	List of external input nodes
node_labels.csv	Names of all nodes in the network
specs.txt	Simulation specification file used for this experiment
logic/	Directory containing all logic files associated with this experiment
nodesAvg/	Directory containing activity level information for each node
snapshotAnalysis/	Directory to hold node activity information obtained from multiple points in the course of a simulation
patterns/	Output directory for the patterns extension
bits/	Output directory holding ON/OFF sequences for all nodes in the network

To provide users with the ability to visualize and/or analyze the dynamics of the network of each simulation at the ON/OFF state level of each node, ChemChains saves strings of 1/0 bits for each simulation in text files stored in the 'bits' directory. Each column in each file represents the ON/OFF states for one node (including external inputs) in the network, where '1' codes the ON state and '0' codes the OFF, whereas the rows represent the state of that node in time *t *of a given simulation. Because detailed bit printing of each simulation requires a significant amount of simulation time, bit outputting can be suppressed by using the '-noBits' parameter.

The most notable feature of ChemChains is the ability of users to interact with their models in a continuous manner. Similarly to the external input nodes, the activity of each node is calculated and expressed as a percent ON (as described in the *Boolean networks and their dynamics *section). This allows users of ChemChains to observe activation patterns of all nodes in the network across thousands of simulations with varying stimuli levels. The activity levels of all nodes during each simulation can be found in a tabular file ('allNodes_avg.csv'). To provide users with detailed experiment information, the 'nodesAvg' directory contains separate files with activity levels (and the values of external inputs that resulted in the node's activity) for each node in the network. ChemChains also allows users to capture "snap shots" of the network's dynamics in different time points of a single simulation. This feature can be enabled in the simulation specification file as described in the *Simulation specification file *section. The snapshot activity information obtained about the network is subsequently saved in the 'snapshotsAnalysis' directory.

In addition, the calculation mode allows users to perform various analyses after each simulation. These analyses are implemented as separate extensions to the software, and can be instantiated using various parameters when starting new experiments. The current version of ChemChains contains the 'Patterns' extension used in our recent study [14]. Description of this extention follows.

#### Patterns extension

ChemChains can be used to conduct experiments consisting of thousands of simulations under various input conditions (including any number of mutations), generating a large amount of data ready for systems analysis. The patterns extension allows users to view the system's dynamics in a more organized and intuitive manner by classifying the activity levels of user-defined nodes into groups based on a pre-defined activity level range, called patterns.

For example, consider a single-simulation experiment which results in output node A being 70% ON, node B 2% ON, and node C 23% ON. To create a pattern representing the activity of these nodes as the outcome of the simulation, the percentage ON of individual output nodes is classified as one of three levels (note that these levels have been chosen arbitrarily for this example, and can be changed by the user as described later in this section): i) "Low" if the node percent ON is between 0% and 10%, ii) "Medium" for nodes that are ON 11–30%, or iii) "High" nodes with activity between 31% and 100%. Each level is subsequently coded as '0' (low), '1' (medium), and '2' (high). Therefore, the pattern for output nodes A, B, and C is '201'.

**Figure 5:**
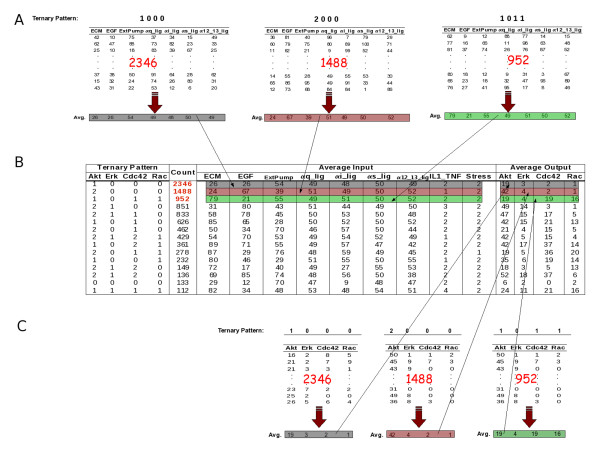
**An example of pattern creation**. Panel B is a summary table of the output data generated by the Patterns extension. The leftmost part of this table contains patterns generated throughout the experiment, whereas the "Count" column represents the number of simulations whose output nodes resulted in the particular pattern, as described in the main text (for easier exemplification, only patterns that occurred more than 100 times are listed in the table). The next two sections of this table contain the average input and average output vectors, respectively. Panel A illustrates the process of creating average input vectors of the top three patterns; that is, for each pattern, the average of external-input dosages of all simulations that were assigned to the particular pattern is calculated. Similarly, the average output vectors are calculated by averaging the output node activity levels across simulations that led to a given pattern. As shown in Panel C, the top three average output vectors (and patterns) were calculated from 2,346, 1,488, and 952 simulations, respectively.

When performing ChemChains experiments consisting of multiple simulation, each simulation will be assigned to a pattern based on the outcome of individual output nodes, as explained above. Simulations assigned the same patterns will form groups represented by the pattern. Each pattern is then associated with an average input vector and average output vector (Figure 5B). Figure 5 illustrates how the output of the Patterns extension is created using partial data generated from a ChemChains experiment on a large-scale model used in our recent paper [14]. The external input nodes of the large-scale model used include Extracellular Matrix (ECM), Epidermal Growth Factor (EGF), External Calcium Pump (ExtPump), four G-Protein Coupled Receptor ligands (*α*q, *α*s, *α*i, and *α*1213), IL1/TNF, and extracellular stress, whereas the output nodes of interest are Akt, Erk, Cdc42, and Rac. The subsequent experiment consisted of 10,000 simulations with randomly generated combinations of external input node dosages during which the activity (percentage ON) of the four aforementioned output nodes was measured. See Figure 5 caption for more details demonstrating how the multiple simulation output is created.

Because the ranges defining each activity level (e.g., low, medium, high), as well as the number of levels used in the example are arbitrary, users can define these properties according to their needs in the 'inFiles/exts/Patterns/settings.txt' text file. The patterns output nodes are defined (one node per line) in the 'inFiles/exts/Patterns/patternsOutNodes.txt' file. These patterns consist of the defined output nodes, whose activity reaches levels within a user-specified range over multiple simulations, as well as the input sets that result in these levels, allowing users to assess global-input vs. global-output relationships. The summary results (average patterns) from the pattern analysis extension are saved by ChemChains in a tab-delimited output file, while the detailed data (*i.e*., simulations with the activity levels of each output and input node, that belong to each pattern) are stored in a spreadsheet file created in a folder named after the pattern, in the 'patterns' folder for later revisions of the experiment data.

## Results and discussion

Modeling of biological processes has become an important component of biomedical research that provides additional tools to study and understand the underlying mechanisms of these processes. The biological modeling community has made significant progress in developing a number of sophisticated mathematical tools and methods that provide the ability to create models and view their dynamics. However, with the increasing sophistication of the mathematics underlying the representation and simulation of various biological systems, only investigators who are trained modelers can use and develop such systems; it essentially becomes impossible for laboratory scientists to create and use sophisticated models directly.

Due to the complex mathematics and the immense number of parameters required, the practical use of continuous models and simulation tools as a daily part of laboratory research is difficult. Although other approaches based on less mathematical concepts such as Petri Nets (which uses a weighted firing approach) have been used for modeling of biological processes [21,22], Boolean modeling combines the mathematical simplicity of those methods with a more intuitive way of interpreting the subsequent models. On the other hand, the inherent discrete nature of logical models does not square with the fact that laboratory experiments are performed in terms of continuous data (e.g., specifying levels of ligands, proteins activity, etc.). This creates an interpretation barrier between the data output of discrete tools and the experiments laboratory biologists conduct. In other words, it would be best for biologists if their *in silico *experiments produced richer data than 0's and 1's. To address this issue, ChemChains allows users to interact with their Boolean models in a continuous fashion, while preserving all advantages of logical modeling. Users have the ability to specify the activity of their external input nodes (representing various network stimuli) as a percentage ON. Similarly, the activity levels of all (or user-selected) nodes are expressed as percent ON. As discussed in the *Implementation *section, the "percent ON" is a way to represent an attractor in Boolean models, and the biological meaning of the percent ON measure is dependent on the nature of the nodes and the overall model. For example, it might correspond to the level (or probability) of phenotype manifestation such as differentiation into a particular cell type or, as in our signal transduction model from our recent study [14], percent ON could correspond to the ability of a signaling molecule (e.g., protein) to transmit a signal received from another signaling molecule to a downstream effector molecule. Depending on the nature of the signaling molecule, that ability might be expressed in terms of protein activity, expression, concentration, etc. The advantage of the percent ON measure used in ChemChains is that it provides a semi-quantitative way to measure the levels of the nodes' activity and/or the direction of a change in these levels due to perturbations, something that laboratory scientists deal with on daily basis and hence can find intuitive and relatively easy to use.

In addition to the above mentioned functionality that makes *in silico *simulations of Boolean models more accessible to laboratory scientists, ChemChains also offers powerful analysis that can be performed by running automated experiments in which users can simulate their models under tens of thousands of randomly generated external conditions. The available Patterns extension categorizes each simulation and a specified group of output nodes into discrete groups, representing global responses (patterns) based on their activity level. Each pattern is then represented by an average output vector and an associated average (external) input vector, which provides a quick way for biologists to learn how their model responds to certain a set of stimuli and how these stimuli map to a specific response; an important feature to aid the understanding of how complex biological structures function and improve the development (and efficiency) of treatments for diseases associated with these systems [23]. Furthermore, investigators can use the software output to easily verify how well the activity levels of specified nodes fit the available empirical laboratory data (e.g., the effects of receptor activation on the activity of a protein in a different area of the network). This presents to biologists a novel tool to obtain insight about unavailable regulatory information, which can be confirmed or rejected by a controlled experiment in the laboratory.

Table 5 shows output produced by the Patterns extension after a sample simulation experiment performed on a Boolean model representing a signal transduction network in a generic fibroblast cell. The sample experiment consisted of 1,000 simulation with randomly selected dosage values for external inputs. The simulated model was introduced in our recent publication [14], and consists of three main pathways; the Tyrosine Receptor Kinase, Integrin, and G-protein Coupled Receptor pathways (and comprises of about 135 nodes and 800 connections). The external input nodes (that make up the average input vector) in Table 5 represent ligands associated with these pathways, while the output nodes (nodes of interest) make up the average output vector, providing biologists with the aforementioned important input-output mapping information. An example of a real biological application of ChemChains and the Patterns extention is our recent study in which the use of these tools in the combination with the above mentioned large-scale Boolean model of biochemical signal transduction network revealed important information about the role of signal transduction networks as information processing and decision-making machinery of the cell [14].

**Table 5:** Sample output generated by the Patterns extension.

		**Average Inputs**									**Average Outputs**			
		
**Global Output**	**Count**	**ECM**	**EGF**	**ExtPump**	**alpha_q Ligand**	**alpha_i Ligand**	**alpha_s Ligand**	**alpha_12_13 Ligand**	**IL1_TNF**	**Stress**	**Akt**	**Erk**	**Cdc42**	**Rac**
1000	218	23	26	55	51	50	45	55	2	2	20	3	1	1
2000	157	23	67	38	45	47	51	50	1	2	43	4	2	1
2100	96	29	78	40	50	44	44	54	4	2	48	14	3	2
2110	96	60	76	48	48	55	53	48	2	2	47	15	17	6
1011	77	73	21	55	46	60	50	53	2	2	20	5	18	15
2111	62	85	68	34	54	53	49	58	2	2	43	16	21	13
2010	46	54	65	54	47	50	52	45	1	2	40	5	15	5
1010	41	49	30	66	43	48	52	46	2	2	20	4	15	6
2121	37	88	70	54	54	58	48	41	2	1	41	17	38	14
1021	35	88	26	77	47	55	57	33	2	2	19	5	36	21
2011	29	84	50	40	54	53	63	54	1	2	35	6	19	15
1001	16	69	18	40	59	25	50	54	2	1	18	4	5	12
0000	13	45	10	49	47	4	54	54	2	2	6	3	0	5
1121	12	83	40	60	46	41	48	31	2	2	24	12	40	17
2120	10	69	82	71	46	57	55	37	2	1	49	16	34	6
1111	8	82	30	29	60	41	53	38	3	2	25	10	18	16
1022	7	97	7	85	48	64	18	39	2	2	13	4	40	33
2021	6	87	62	56	62	57	53	51	1	3	38	6	33	14
1110	5	55	56	72	58	51	55	38	4	2	26	12	16	6
1020	5	62	46	87	51	24	43	18	2	1	20	5	32	8
1100	4	29	54	68	46	29	44	44	4	2	22	11	2	2
2200	4	38	95	74	46	36	36	54	4	3	67	34	4	3
2210	3	67	95	72	45	41	20	52	4	2	62	31	23	5
0001	3	79	10	87	64	9	65	73	3	0	8	4	5	15
0011	3	78	14	92	49	22	74	67	0	3	8	0	18	18
2221	2	92	91	54	11	49	12	54	4	1	58	30	38	12
2020	2	70	89	98	54	34	3	46	0	3	65	3	46	6
2220	1	71	99	77	67	63	27	16	5	5	55	33	33	8
1101	1	89	40	18	31	1	68	22	5	5	27	12	7	14
2101	1	93	79	66	75	0	77	97	4	1	53	22	7	12

In this particular experiment, ECM, EGF, ExtPump, alpha_q, alpha_i, and alpha_12_13 ligands were set to vary between 0–100% ON, while IL1_TNF and Stress varied between 0 and 5% ON. The first and second columns summarize all global outputs (or patterns) and their frequency produced by the model in response to the various external stimuli. Columns labeled as Average Inputs represent all external inputs and their average % ON value for a given global output. Similarly, Average Outputs contain output nodes and their average % ON for a given pattern. In this example the patterns are in ternary: 0–9% ON = 0, 10–29% ON = 1, and 30–100% ON = 2. These ranges were determined to be the most useful in previous experiments [14], but any ranges (or discretization) can be used. Focusing on the first row, it can be seen that there were 218 of the 1000 simulations where output pattern was '1000', i.e., Akt output was in the 10–29% range, Erk was in the 0–9% range, etc. Of those 218 simulations, the average output values of Akt was 20, the average Erk value was 3, etc. The average external-input values that elicited the '1000' response is given in each of the Inputs columns, e.g., the average external-input value that resulted in the '1000' response was ECM = 23% ON, EGF = 26% ON, etc. These experiments were carried out under non-stress conditions, thus IL1_TNF and Stress external inputs varied only from 0 to 5% ON.

Another ChemChains function of great use for many laboratory scientists is the ability to perform *in silico *mutational studies. ChemChains offers the ability to select nodes that will simulate the loss-of-function or gain-of-function scenarios (which can be specified in the simulation specification file provided to ChemChains prior to new simulations). As an example of how ChemChains, in combination with the Patterns extension, can be used for the above mentioned mutation studies is illustrated on the signal transduction model (mentioned above) in Table 6. In this example, Ras (a protein often responsible for some types of cancer) was "mutated" to stay ON, which resulted in a significant activity increase of Erk (a cell growth regulatory component, [24,25]) as well as the anti-apoptosis Akt, a possible indication of uncontrolled cell growth. This corresponds to a cancerous cell, whose signaling has been altered. This type of input-output mapping allows biologists to predict the behavior of the model in response to its stimuli.

**Table 6:** Sample experiment output with constitutively active Ras.

		**Average Inputs**									**Average Outputs**			
		
**Global Output**	**Count**	**ECM**	**EGF**	**ExtPump**	**alpha_q Ligand**	**alpha_i Ligand**	**alpha_s Ligand**	**alpha_12_13 Ligand**	**IL1_TNF**	**Stress**	**Akt**	**Erk**	**Cdc42**	**Rac**
2200	229	39	62	37	51	50	49	51	2	2	49	79	4	3
2211	172	83	46	42	46	48	45	52	2	2	42	75	18	15
2210	153	55	69	53	51	52	51	49	2	2	48	82	15	5
1000	142	19	23	64	51	46	47	45	2	2	19	3	1	1
2000	75	11	62	44	54	54	50	56	1	2	42	3	0	0
1211	45	87	24	71	48	46	53	54	2	2	23	63	16	19
2100	32	13	78	61	49	51	48	51	3	2	49	15	1	0
2201	28	83	46	22	46	56	51	57	2	2	46	76	6	14
1200	25	41	27	77	48	50	43	47	2	2	22	61	5	4
1201	21	75	9	65	57	48	46	47	2	2	21	58	5	17
1210	16	54	37	83	48	61	38	50	2	2	23	64	14	6
2221	13	89	68	57	43	35	53	49	2	1	46	79	32	15
1001	6	58	11	49	43	26	52	53	1	3	16	1	5	11
2220	5	79	92	86	39	82	33	45	3	2	57	88	32	8
1100	5	32	12	46	45	65	44	57	2	2	22	18	6	4
1011	4	66	13	71	24	33	60	45	2	2	14	2	19	14
1010	4	50	14	88	63	66	46	62	1	2	15	1	16	7
2110	3	36	35	29	31	64	55	55	3	1	34	18	11	5
1212	2	95	4	50	40	65	68	91	2	1	23	56	12	31
1110	2	60	39	84	71	61	26	81	5	3	23	19	19	8
0011	2	67	1	91	54	5	46	74	2	4	8	2	10	17
1202	2	90	5	61	68	53	41	51	2	4	19	57	9	30
1111	2	67	16	100	55	89	80	20	2	3	13	16	24	15
0001	1	71	0	64	68	4	97	69	5	1	8	5	6	17
0100	1	47	16	100	31	13	1	85	3	5	9	15	4	6
0201	1	92	3	100	73	92	85	82	2	5	5	53	6	29
2212	1	99	15	47	59	24	32	17	4	0	34	64	23	30
0010	1	29	20	98	73	7	71	63	1	5	7	1	11	3
1002	1	98	3	58	43	5	59	70	5	3	13	5	9	33
1221	1	100	38	87	95	40	79	51	0	1	23	69	33	26
1021	1	81	23	88	73	10	78	22	1	0	15	1	34	21
0212	1	100	9	99	62	54	19	3	5	1	9	57	17	34
2111	1	50	14	24	41	90	22	32	3	0	31	27	18	10
0202	1	100	4	90	41	49	39	48	5	5	9	55	2	33
0211	1	75	9	96	80	91	95	21	1	3	8	57	11	17

Similarly to Table 5, results summarized in this table were generated during an experiment consisting of 1,000 simulation with the same paremeters (e.g., all dosages were selected randomly in a stress limited environment, discretization, *etc*.). Contrary to the simulation from Table 5 however, in this example, cells were simulated with constitutively active Ras. Results of this experiment show a marked increase in Erk activity (as has been suggested by laboratory research, [24,25]), simulating a growth-factor independent activation of Erk. This phenomena can be easily seen either in the Global Output and Count columns where 229 of the 1,000 simulations produced the '2200' pattern (global output) and over 500 simulations resulted in '22XX' or the Average Output column which shows that the majority of external-input combinations resulted in Erk average activity of 75–82%.

As illustrated by the above examples, ChemChains and its Patterns extension provide biologists with a set of tools that allow predictive model simulations based on the nature of the responses produced as a result of given stimuli (including internal pathological-related conditions such as a cancerous protein mutations). In addition, this software forms a solid platform for laboratory biologists to bring Systems Biology to their laboratory by being able to perform simulations and analysis of model of biological structures directly and independently of mathematicians and modelers.

ChemChains is under constant development to expand its functionality and increase its user-friendliness. The following are examples of areas of ChemChains development our group is addressing or planning to address in the near future. Although the software offers rich functionality while keeping the number of command-line parameters to run the tool low, a graphical user interface would make ChemChains more intuitive and user-friendly. Our group is currently implementing a graphical user interface that will allow laboratory scientists to build logical networks, as well as perform simulation and analysis that will provide users with the means to display their results in a more exible and intuitive way than is currently available. For example, users will be able to control the dosage levels of external inputs via slider controls and observe the activity of output nodes in real time.

Additionally, as new models and simulation tools are created and implemented, respectively, the need for a standard way of sharing model information between various research groups and their simulation tools became apparent. To address this need, M. Hucka et. al. [26] have developed the Systems Biology Markup Language (SBML), a standard describing network models. SBML has been adapted by a large number of simulation tools, allowing users to share their models. Because SBML currently supports mostly continuous modeling techniques, ChemChains is currently not SBML-compatible. However, the SBML developers are currently working on the integration of logical models into the standard for the upcoming version of SBML, therefore the next version of ChemChains is anticipated to include a support for SBML.

Furthermore, to ensure that scientists can use ChemChains for extensive simulations of large-scale models, the software will require the support of multi-core/processor computers. Thus our group is planning on optimizing ChemChains to take advantage of not only multi-core processor computers, but also more powerful supercomputers. This will decrease the amount of time needed to run ChemChains simulations and analysis.

## Conclusion

As systems biology and the development of mathematical models progress and these models become more complex, their use requires more advanced mathematical knowledge. For laboratory biologists to take advantage of the systems biology paradigm as a compliment to their laboratory experiments, simulation tools that will allow them to perform *in silico *simulation experiments in a mathematically friendly fashion is a must. Our group has developed ChemChains, a simulation and analysis platform to allow laboratory scientists to visualize the dynamics of biological processes using non-mathematical, parameter-free logical models. In addition, ChemChains allows laboratory scientists to interact with mathematical models in a way which resembles laboratory experiments, and provides the investigators with new tools to see the big picture in the realm of biological processes. The software allows biologists to perform simulations of their model under thousands of varying stimuli and learn how the models respond to different combinations of conditions, an important step to understanding how many complex biological processes, such as signal transduction, function.

## Availability and requirements

ChemChains has been tested on a Windows and Linux platforms. To run ChemChains on a Windows-based computer, a *nix environment needs to be installed (such as Cygwin [27]) ChemChains software along with a detailed tutorial on how to use the tool are available on-line [28].

## Authors' contributions

TH and JAR designed the software. TH implemented and deployed the software. TH wrote and JAR reviewed the manuscript. Both authors read and approved the final manuscript.
